# A genome-wide assessment of stages of elevational parapatry in Bornean passerine birds reveals no introgression: implications for processes and patterns of speciation

**DOI:** 10.7717/peerj.3335

**Published:** 2017-05-18

**Authors:** Robert G. Moyle, Joseph D. Manthey, Peter A. Hosner, Mustafa Rahman, Maklarin Lakim, Frederick H. Sheldon

**Affiliations:** 1 Department of Ecology and Evolutionary Biology and Biodiversity Institute, The University of Kansas, Lawrence, KS, USA; 2 Department of Biology, University of Florida, Gainesville, FL, USA; 3 Faculty of Natural Science and Sustainability, University College Sabah Foundation, Kota Kinabalu, Sabah, Malaysia; 4 Sabah Parks, Kota Kinabalu, Sabah, Malaysia; 5 Museum of Natural Science and Department of Biological Sciences, Louisiana State University, Baton Rouge, LA, USA

**Keywords:** Parapatry, Speciation, Species pump, Elevation, Borneo, Elevational cline

## Abstract

Topographically complex regions often contain the close juxtaposition of closely related species along elevational gradients. The evolutionary causes of these elevational replacements, and thus the origin and maintenance of a large portion of species diversity along elevational gradients, are usually unclear because ecological differentiation along a gradient or secondary contact following allopatric diversification can produce the same pattern. We used reduced representation genomic sequencing to assess genetic relationships and gene flow between three parapatric pairs of closely related songbird taxa (*Arachnothera* spiderhunters, *Chloropsis* leafbirds, and *Enicurus* forktails) along an elevational gradient in Borneo. Each taxon pair presents a different elevational range distribution across the island, yet results were uniform: little or no gene flow was detected in any pairwise comparisons. These results are congruent with an allopatric “species-pump” model for generation of species diversity and elevational parapatry of congeners on Borneo, rather than in situ generation of species by “ecological speciation” along an elevational gradient.

## Introduction

A common feature of species-rich regions is the spatial association (i.e., sympatry or parapatry) of closely related species (Stuart, Inger & Voris, 2006; Weir & Price, 2011). For allopatric speciation models, regional accrual of species diversity requires a phase of isolation followed by secondary contact and subsequent co-occurrence of recently diverged taxa (Capparella, 1991; Haffer, 1969; Mayr & O’Hara, 1986), and much research has focused on the interaction of recently diverged taxa in lowland habitats (Aliabadian et al., 2005; Lim et al., 2011; Naka et al., 2012). In topographically complex regions, this association of related taxa often takes the form of elevational parapatry between congeners (Chapman, 1926; Diamond, 1972; Terborgh, 1971) that can lead to high beta species diversity in small geographic areas relative to lowland areas (Fjeldså, Bowie & Rahbek, 2012; Myers et al., 2000). The elevational replacement of presumably closely related species has led to much conjecture about the processes that produce and maintain this pervasive pattern (Fjeldså, Bowie & Rahbek, 2012; Hall, 2005; Jankowski, Robinson & Levey, 2010; Päckert et al., 2012; Terborgh & Weske, 1975). Diamond (1973) hypothesized that it could be explained by allopatric divergence of lowland populations, subsequent secondary contact between populations, and elevational displacement of one or both of the taxa via competition.

**Figure 1:**
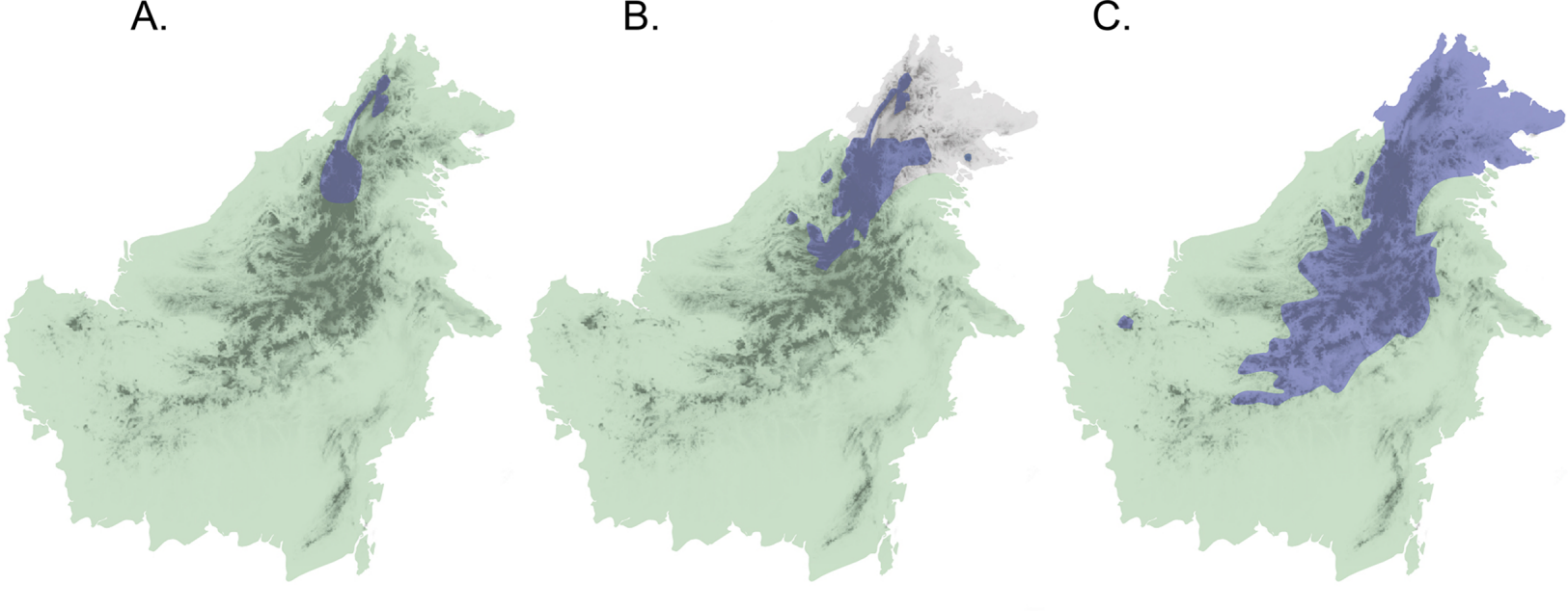
Map of Borneo with approximate distributions of focal species. Blue color denotes the montane representative of the species pair ((A) *Enicurus borneensis*, (B) *Chloropsis kinabaluensis*, and (C) *Arachnothera everetti*); green represents the lowland species ((A) *Enicurus leschenaulti*, (B) *Chloropsis cochinchinensis*, and (C) *Arachnothera modesta*). Note that *Chloropsis cochinchinensis* and *Arachnothera modesta* are absent from Northeast Borneo. Darker shading indicates montane regions.

Application of this secondary-contact model to the Sunda Shelf in Southeast Asia suggests a scenario of isolation and divergence among taxa during periods when land masses (the Sunda Islands) were separated by shallow seas, as at present and during Pleistocene interglacials and in the late Pliocene. Subsequent secondary contact would occur during Pleistocene glacial maxima when sea levels decreased and the Sunda Shelf emerged as a continuous landmass (Salzmann et al., 2011). If Diamond’s model of elevational displacement between recently diverged taxa pertains to these Sundaic montane faunas, we might expect to see pairs of taxa in various stages of elevational displacement, due to variation in opportunities for population differentiation and expansion. In fact, the island of Borneo contains several avian examples of species or populations that appear to be in different stages of secondary contact with incipient or recent elevational displacement (Sheldon, Lim & Moyle, 2015). Three species complexes (*Chloropsis cochinchinensis*, *Enicurus leschenaulti*, and *Arachnothera everetti*) occur widely in lowland and lower montane habitats in Sundaland, extending into Southern mainland Asia to differing degrees. Each complex contains a single taxon on Sumatra and Java, but two taxa on Borneo that display elevational segregation over part or all of their distributions (Fig. 1). Members of both the *Chloropsis cochinchinensis* and *Arachnothera everetti* complexes are partially segregated elevationally, with distinct montane (*Chloropsis kinabaluensis* and *Arachnothera everetti*) and lowland (*Chloropsis cochinchinensis* and *Arachnothera modesta*) taxa in central Borneo, but with only the montane taxa occurring in Northeast Borneo (Fig. 1). Complete elevational segregation of populations across all of the higher montane areas of Borneo apparently occurs in the *Enicurus leschenaulti* complex, with *Enicurus leschenaulti* in the lowlands and *Enicurus borneensis* in the mountains (Fig. 1).

However, the taxonomic status, distribution, and interaction between these pairs of taxa on Borneo have been a source of contention. Morphological differences within each pair are slight (although female plumage in *Chloropsis kinabaluensis* is quite distinct). Indeed, the taxa in each of the three focal pairs have often been considered conspecific, with multiple parapatric subspecies occurring in Borneo (BirdLife International, 2015; Dickinson, 2003; Inskipp, Lindsey & Duckworth, 1996; Smythies, 1957). Phylogenetic studies using mitochondrial DNA sequences (Moltesen et al., 2012; Moyle et al., 2005, 2011) indicate that members of each montane–lowland pair are closely related (<5% divergence), and that the lowland populations from Western Borneo are part of clades that extended to Sumatra (and the Malay Peninsula if sampled). Javan populations are either part of the lowland radiation (*Chloropsis*; Moltesen et al., 2012), sister to the rest of the complex (*Arachnothera*; Moyle et al., 2011), or unresolved with respect to the Bornean lineages (*Enicurus*; Moyle et al., 2005). Limited character and taxon sampling in previous studies precluded inferences about interaction or evolutionary independence between members of each pair (Moltesen et al., 2012; Moyle et al., 2005, 2011). Shifting opinions on species concepts have recently caused each complex to be recognized as multiple species by some authors (Gill & Donsker, 2015), but interactions between these recently derived populations are essentially unknown, and some researchers have surmised that the parapatric taxa might instead represent an elevational cline (Collar & Pilgrim, 2007).

As a next step in deciphering the relationships within these three species complexes, we used restriction site associated DNA sequencing (RAD-seq) and an east–west transect of geographic and elevational sampling to assess the population structure and potential gene flow between the three pairs of elevationally segregated bird populations on Borneo. For simplicity, hereafter we use the classification of Gill & Donsker (2015), which assigns each population to species status.

## Materials and Methods

### Sampling, laboratory procedures, and SNP dataset creation

More than 15 years of field work in Malaysian Borneo (permits from Malaysian Prime Minister’s Department, UPE: 40/200/19 SJ.1039 and UPE: 0/200/19/2401; Approved IACUC protocol: 174-01) has resulted in dense geographic sampling of birds across the northern tier of the island, including most of the higher mountains, such as Kinabalu, Trus Madi, and Mulu (Burner et al., 2016; Moyle & Wong, 2002; Sheldon et al., 2009). Because standard RAD-seq does not typically perform well with low-molecular weight historical DNA samples (i.e., degraded DNA from museum skins), we used only DNA extracted from fresh tissue samples.

Ethanol-preserved tissue samples of the six species representing high- and low-elevation taxa, respectively, were sampled from Malaysian Borneo: *Arachnothera everetti*, *Arachnothera modesta*, *Chloropsis kinabaluensis*, *Chloropsis cochinchinensis*, *Enicurus borneensis*, and *Enicurus leschenaulti*. Because canopy species are more difficult to capture with mist-nets than understory species, sampling of individuals in *Chloropsis* was notably sparser than in *Arachnothera* and *Enicurus* (Table 1; an additional *Chloropsis* from Singapore was sampled).

**Table 1:** Samples included in study, including museum voucher number, geographic coordinates, sampling elevation (m), number of raw sequencing reads, and percent coverage in the final dataset (Cov.).

Species	Number	Locality	Latitude	Longitude	Elevation	# Reads	Cov. (%)
*Arachnothera everetti*	KU-17782	Sabah, Ulu Kimanis	5°30.28′N	116°00.79′E	550	197,372	58
*Arachnothera everetti*	KU-17801	Sabah, Mt. Kinabalu	6°02.12′N	116°33.02′E	2,100	615,882	71
*Arachnothera everetti*	LSU-36310	Sabah, Crocker Range	5°23′59″N	116°06′08″E	1,000	517,039	95
*Arachnothera everetti*	LSU-38631	Sabah, Sayap	6°10′N	116°34′E	950	246,648	88
*Arachnothera everetti*	LSU-38634	Sabah, Sayap	6°10′N	116°34′E	950	878,430	90
*Arachnothera everetti*	LSU-38648	Sabah, Sayap	6°10′N	116°34′E	950	262,400	83
*Arachnothera everetti*	LSU-38655	Sabah, Sayap	6°10′N	116°34′E	950	1,433,139	93
*Arachnothera everetti*	LSU-38661	Sabah, Sayap	6°10′N	116°34′E	950	238,997	86
*Arachnothera everetti*	LSU-47093	Sabah, Serinsim	6°17′36″N	116°42′30″E	200	98,415	41
*Arachnothera everetti*	LSU-47125	Sabah, Serinsim	6°17′36″N	116°42′30″E	200	237,231	78
*Arachnothera everetti*	LSU-50996	Sabah, Mendolong	4°51′N	115°42′E	1,100	441,833	93
*Arachnothera everetti*	LSU-51037	Sabah, Mendolong	4°51′N	115°42′E	1,100	378,029	94
*Arachnothera everetti*	LSU-52653	Sabah, Mt. Trus Madi	5°35′N	116°29′30″E	1,450	733,236	73
*Arachnothera everetti*	LSU-61591	Sabah, Ulu Kimanis	5°30′N	116°01′E	550	402,149	82
*Arachnothera everetti*	LSU-61619	Sabah, Ulu Kimanis	5°30′N	116°01′E	550	196,124	79
*Arachnothera everetti*	LSU-78712	Sarawak, Kelabit Highlands	3°48′N	115°28′E	1,150	477,894	95
*Arachnothera everetti*	LSU-78714	Sarawak, Kelabit Highlands	3°48′N	115°28′E	1,150	636,850	79
*Arachnothera everetti*	LSU-78744	Sarawak, Kelabit Highlands	3°48′N	115°28′E	1,150	701,378	96
*Arachnothera modesta*	LSU-52174	Sarawak, Kuching	1°37′N	110°12′E	75	721,231	97
*Arachnothera modesta*	LSU-79469	Sarawak, Mt. Pueh	1°43′N	109°43′E	40	129,136	73
*Arachnothera modesta*	LSU-79500	Sarawak, Mt. Pueh	1°08′N	110°13′E	750	353,643	97
*Arachnothera modesta*	LSU-79512	Sarawak, Mt. Pueh	1°08′N	110°13′E	750	184,233	55
*Arachnothera modesta*	LSU-79540	Sarawak, Mt. Pueh	1°08′N	110°13′E	750	751,892	92
*Arachnothera modesta*	LSU-79587	Sarawak, Mt. Pueh	1°08′N	110°13′E	750	127,247	52
*Arachnothera modesta*	LSU-79624	Sarawak, Mt. Pueh	1°08′N	110°13′E	750	160,778	81
*Arachnothera modesta*	LSU-84875	Sarawak, Singai	1°30′N	110°10′E	90	1,248,393	73
*Chloropsis cochinchinensis*	AMNH-9638	Singapore				823,087	26
*Chloropsis cochinchinensis*	LSU-84885	Sarawak, Singai	1°30′N	110°10′E	90	608,352	73
*Chloropsis kinabaluensis*	LSU-52618	Sabah, Mt. Trus Madi	5°34′N	116°29′E	1,650	167,501	74
*Chloropsis kinabaluensis*	LSU-52620	Sabah, Mt. Trus Madi	5°34′N	116°29′E	1,650	404,241	88
*Chloropsis kinabaluensis*	LSU-52621	Sabah, Mt. Trus Madi	5°34′N	116°29′E	1,650	322,979	93
*Chloropsis kinabaluensis*	LSU-52685	Sabah, Mt. Trus Madi	5°35′N	116°29′E	1,450	705,094	91
*Enicurus borneensis*	KU-17795	Sabah, Mt. Kinabalu	6°00.34′N	116°32.55′E	1,550	637,728	52
*Enicurus borneensis*	LSU-36442	Sabah, Mt. Trus Madi	5°35′N	116°29′30″E	1,500	2,415,758	98
*Enicurus borneensis*	LSU-36452	Sabah, Mt. Trus Madi	5°35′N	116°29′30″E	1,500	829,179	67
*Enicurus borneensis*	LSU-52604	Sabah, Mt. Trus Madi	5°35′N	116^o^29′E	1,450	162,015	10
*Enicurus borneensis*	LSU-61641	Sabah, Mt. Kinabalu	6°00′N	116°32′30″E	1,600	2,429,951	98
*Enicurus borneensis*	LSU-61642	Sabah, Mt. Kinabalu	6°00′N	116°32′30″E	1,600	2,357,570	87
*Enicurus borneensis*	LSU-78706	Sarawak, Kelabit Highlands	3°48′N	115°28′E	1,150	1,250,182	75
*Enicurus leschenaulti*	LSU-38580	Sabah, Tawau Hills	4°24′N	117°54′E	250	1,159,426	45
*Enicurus leschenaulti*	LSU-38581	Sabah, Tawau Hills	4°24′N	117°54′E	250	654,566	28
*Enicurus leschenaulti*	LSU-47113	Sabah, Serinsim	6°17′36″N	116°42′29″E	200	2,049,467	98
*Enicurus leschenaulti*	LSU-47120	Sabah, Serinsim	6°17′36″N	116°42′29″E	200	3,502,526	95
*Enicurus leschenaulti*	LSU-47134	Sabah, Serinsim	6°17′36″N	116°42′29″E	200	791,251	34
*Enicurus leschenaulti*	LSU-51050	Sabah, Tawau Hills	4°24′N	117°53′E	250	2,090,691	94
*Enicurus leschenaulti*	LSU-52234	Sarawak, Bintulu	2°54′N	112°52′E	200	2,605,395	86

**Note:**

Museum abbreviations: KU, The University of Kansas Natural History Museum and Biodiversity Institute; LSU, Louisiana State University Museum of Natural Science; AMNH, American Museum of Natural History.

Total genomic DNA was extracted using a QIAGEN DNeasy blood and tissue extraction kit following manufacturer protocols. We performed a modified restriction-site associated DNA sequencing (RAD-seq; Miller et al., 2007) protocol to obtain a reduced representation genomic library. All samples were digested with the restriction enzyme *Nde*I. Subsequently, we ligated custom adapters with unique barcodes to all samples for multiplexing. Following barcode ligation, all samples were pooled and then purified using AMPure magenetic beads (Agencourt). We further reduced the library by size-selecting fragments of length 500–600 bp using a Pippin Prep electrophoresis cassette (Sage Science, Beverly, MA, USA), trailed by another round of DNA purification. Lastly, we performed a PCR of the library in quadruplicate using an initial denaturation period of 98 °C for 30 s, 14 cycles of 98 °C for 10 s, 64 °C for 30 s, and 72 °C for 20 s, and a final extension of 72 °C for 7 min. The library was tested for DNA quality and quantity using quantitative PCR and the Agilent TapeStation at The University of Kansas Genome Sequencing Core Facility, followed by sequencing of 100 bp single-end reads on a partial lane of an Illumina HiSeq2500.

To create a SNP library from the Illumina sequence data, we used the STACKS pipeline (Catchen et al., 2011), and its included modules: *process_RADtags*, *ustacks*, *cstacks*, *sstacks*, and *populations*. Sequences were removed if they contained a 15 bp window with an average Phred score less than 10, contained possible adapter contamination, or lacked the restriction site. We used default parameters in *ustacks*, *cstacks*, and *sstacks*, with the exception that we allowed five mismatches between individuals when creating loci in cstacks (changed from the default of two). In the *populations* module, we selected SNPs with the following criteria: (1) present in a minimum of 50% of individuals of each taxon (see Table 1) and (2) a minimum stack depth at each locus of five. We used a minimum minor allele frequency of 5%. To reduce inclusion of possible paralogous loci, we removed loci with observed heterozygosity greater than 50% or with excessive polymorphism (outside the distribution of SNPs seen in Figs. S1–S3).

To create a more stringent SNP matrix, we tested each taxon’s SNP dataset for possible selection by using BayeScan v2.1 (Foll & Gaggiotti, 2008). Briefly, BayeScan compares the posterior probability of a neutral model based on a population-level measure of genetic differentiation to the posterior probability of a selection model that incorporates locus-specific measures of genetic differentiation to explain alternative allele frequencies between populations. We ran BayeScan for 20 initial pilot runs followed by a final run with 50,000 burn-in steps and 50,000 iterations sampled every 10. We used default settings as implemented in BayeScan as a liberal search for loci under selection (i.e., possible high false discovery rate). With the results, we interpreted the log posterior odds using Jeffrey’s scale of evidence for Bayes factors (Jeffreys, 1961), where values above one are considered evidence for selection. With these tests, in all three taxa, no SNPs showed evidence of selection.

We used the BLAST+ utility (Camacho et al., 2009) to match all loci recovered from each species group to chromosomes on the Zebra Finch (*Taeniopygia guttata*) genome. Sequences were paired to the best matching chromosome if they contained a minimum of 70% sequence identity with the Zebra Finch, and the *e*-value of the match was below 0.01. To remove loci that were in potential physical linkage, we used the BLAST+ results to exclude loci that were within 10,000 bp of each other based on the Zebra Finch genome.

To assess the impact of changing the number of mismatches allowed between individuals when creating stacks (*cstacks* module), we varied the parameter *N* between values of two and seven for each of the three taxa while keeping all other settings as described above (exclusion of physical linkage and selection tests). With these datasets, we investigated patterns of genetic differentiation (*F*_ST_), polymorphisms, and genetic structure (using STRUCTURE [Pritchard, Stephens & Donnelly, 2000] as described below). Similarly, we also changed the minimum stack depth (*m*) in the STACKS *populations* module (with *N* = 5) and investigated how this influenced results of differentiation, polymorphisms, and genetic structure. In *Arachnothera*, changing *N* or *m* minimally affected any of these downstream analyses (Figs. S4–S6). Similarly, changing *N* had little effect on downstream analyses in *Enicurus* (Figs. S4, S7 and S8), whereas STRUCTURE analyses showed less power to assign a couple of individuals to their respective genetic clusters (Fig. S8) when the minimum stack depth was high (*m* = 15), likely because the analysis was limited to an order of magnitude fewer SNPs. In contrast to *Arachnothera* and *Enicurus*, the *Chloropsis* dataset indicated that changing the value of *N* influenced the results (Figs. S4, S9 and S10); increasing *N* yielded higher *F*_ST_ (Fig. S4) values between populations and larger proportions of fixed differences relative to private polymorphisms (Fig. S9), suggesting that increasing the value of *N* allowed the merging of more loci with fixed differences between the two lineages.

### Population structure, migration, and population sizes

To infer population structure from the SNP data, using a single SNP per locus (selected randomly), we used the program STRUCTURE (Falush, Stephens & Pritchard, 2003; Pritchard, Stephens & Donnelly, 2000). For each species group, we inferred lambda by estimating the likelihood of one population (*k* = 1), and allowing lambda to converge. All subsequent STRUCTURE runs used this fixed value for lambda (from the initial run as suggested by the STRUCTURE manual), correlated allele frequencies, and the admixture model. For each SNP dataset, we implemented five replicate 150,000 MCMC generation STRUCTURE runs for *k* = 1–3; the first 50,000 generations were discarded as burnin. To identify the most likely number of populations, we used the Δ*K* method (Evanno, Regnaut & Goudet, 2005).

To generate posterior probability distributions of the population demographic parameters Theta (θ; 4 Nμ) and *M* (m/μ), we used MIGRATE-N v3.6 (Beerli, 2006; Beerli & Felsenstein, 2001). Migrate-N analyses used all SNPs; however, they were merged into the consensus reads, so that input for the analyses was each RAD locus, with the polymorphic sites inserted. For each dataset, we estimated Theta for each population (as inferred by STRUCTURE), and estimated migration between populations. We performed three replicate runs of Migrate-N, using empirical estimates of *T*_I_/*T*_V_ and base frequencies (calculated using MEGA v.5.2; Tamura, Nei & Kumar, 2004; Tamura et al., 2011), default settings, and exponential priors on Theta and *M*. Each MCMC chain was run 2,000,000 steps, sampled every 100 steps; samples representing the first 1,000,000 steps were discarded as burnin. We assessed chain mixing by examining acceptance ratios and effective sample sizes of all parameters and genealogies. We evaluated convergence by examining all parameter estimates from independent runs.

For each of the population pairs, we used the program *∂a∂i* (Gutenkunst et al., 2009) to test multiple divergence scenarios: (1) no population split, (2) strict isolation following divergence, and (3) isolation with migration following divergence. In *∂a∂i*, we tested different models utilizing a composite log-likelihood-based multinomial approach using site frequency spectra (Gutenkunst et al., 2009). As input for *∂a∂i*, we used SNP matrices for each population pair that included one biallelic SNP per locus, and included in a minimum of 12, 4, or 8 alleles for each population in *Arachnothera*, *Chloropsis*, and *Enicurus*, respectively. These matrices were used to derive a site frequency spectrum of all SNPs based on minor allele frequencies (folded terminology in *∂a∂i*). All demographic modeling in *∂a∂i* was projected down to the minimum alleles per population. For each divergence scenario, we performed three replicates. Based on an inability to obtain consistent results for divergence scenarios in *Chloropsis*, possibly due to small sample size, we omitted further inclusion of this taxon in *∂a∂i* analysis.

## Results

### Properties of sequence data and SNP datasets

**Figure 2:**
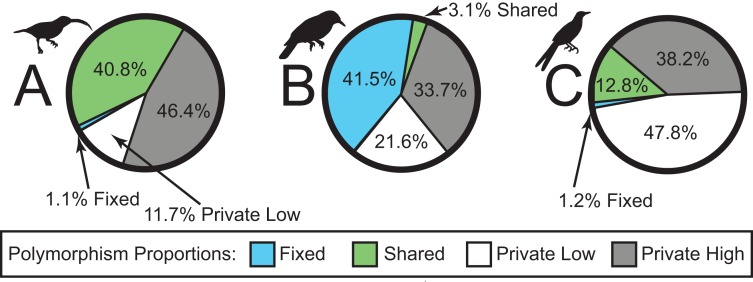
Sources of genetic variation in (A) *Arachnothera*, (B) *Chloropsis*, and (C) *Enicurus*. A high proportion of polymorphisms was restricted to either high- or low-elevation populations (gray and white sections). A large proportion of polymorphism is identified as fixed between *Chloropsis* lineages.

We sequenced a total of 46 individuals on a partial Illumina HiSeq2500 lane, obtaining a total of 38,336,558 sequence reads. The number of sequence reads per individual was highly variable, with a mean of 833,403 (98,415–3,502,526; median = 626,366; standard deviation = 798,974). In total, sequencing yielded a ∼3.45 billion bp. The SNP datasets consisted of 2,791, 1,190, and 1,910 loci for *Arachnothera*, *Chloropsis*, and *Enicurus*, respectively (Table 2). Based on BLAST+ searches, coverage was consistent across chromosomes for all species (Table 2). The majority of SNPs varied only in one lineage (high- or low-elevation groups) for each species (Fig. 2). The *Chloropsis* dataset had a large proportion of fixed differences between high- and low-elevation birds relative to other species (Fig. 2). To test if the high proportion of fixed differences in *Chloropsis* resulted from small sample sizes, we examined polymorphism ratios of subsampled datasets in both *Arachnothera* and *Enicurus*. We performed 100 replicates of randomly sampling four highland and two lowland individuals in each taxon (i.e., equivalent to the *Chloropsis* sampling) and summarized the polymorphisms. The level of fixed differences identified in *Chloropsis* was never attained in the other two taxa in the randomized subsamples (Fig. S14), suggesting that sample size alone did not drive the high proportion of fixed differences in *Chloropsis*.

**Table 2:** Summary by species group.

Group	*N* low	*N* high	# Loci	# SNPs	*T*_I_/*T*_V_	*F*_ST_	Loci/Chr.
*Arachnothera*	8	18	2,791	4,856	4.30	0.135	0.983 (*p* < 0.001)
*Chloropsis*	2	4	1,190	2,455	6.60	0.522	0.964 (*p* < 0.001)
*Enicurus*	7	7	1,910	3,260	3.37	0.117	0.972 (*p* < 0.001)

**Note:**

Sample size of high and low elevation individuals (*N* low or high), number of polymorphic loci (# loci), number of SNPs (# SNPs), transition to transversion ratio used for Migrate-N analyses (*T*_I_/*T*_V_), overall *F*_ST_ between high- and low-elevation groups, and relationship (*R*^2^) of number of loci and chromosome size (Loci/Chr.).

### Phylogeographic structure and demographic estimates

**Figure 3:**
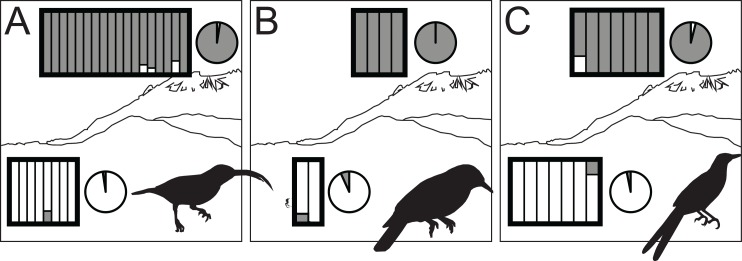
STRUCTURE results for highland (dark gray) and lowland (white) species of *Arachnothera* (A), *Chloropsis* (B), and *Enicurus* (C). In the rectangles, each vertical bar indicates assignment probability for an individual to the highland or lowland genetic groups. Pie charts indicate proportion of overall highland or lowland assignment.

**Figure 4:**
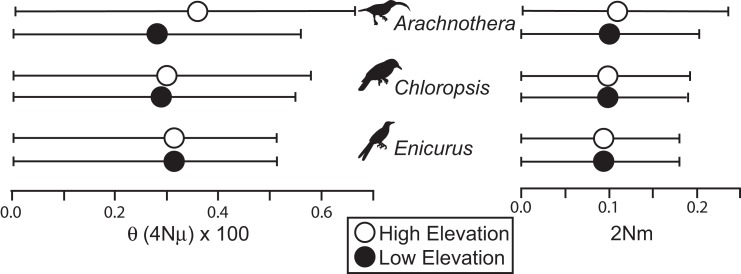
Results of Migrate-N coalescent-based demographic analyses. Shown are estimated mean (dots) and associated 95% CI for population size (θ) and gene flow into a population (2 Nm).

All species showed moderate to high *F*_ST_ values between high- and low-elevation groups (0.11–0.52; Table 2). STRUCTURE analyses identified the number of genetic clusters to be two for each congeneric pair of taxa (*k* = 2; Figs. S4–S6). Support in assigning each individual to a genetic cluster was generally high (Fig. 3), with little evidence of admixture between groups. Coalescent-based demographic analyses performed in Migrate-N indicated very low estimates of gene flow (2 Nm ≪ 1) and highly overlapping estimates of θ (4 Nμ; Fig. 4). Results among replicate runs of Migrate-N were qualitatively very similar (Table S1). Effective MCMC sample sizes and acceptance ratios for all parameter estimates were greater than four million and 0.24, respectively. For both *Arachnothera* and *Enicurus*, isolation with migration was the best divergence scenario tested in *∂a∂i* (Table S2). In each case, the level of gene flow between population pairs was low (2 Nm < 0.5).

## Discussion

Genome-wide assessment of relationships and genetic characteristics of closely related, elevationally parapatric congeners in Borneo indicated low or nonexistent levels of gene flow. Because of extensive presumed contact in segments of the ranges of *Chloropsis* and *Arachnothera* spp., and parapatry across most of the range of *Enicurus* spp., the genetic distinctiveness of these populations supports a lack of, or extremely restricted introgression between the taxa. Because we did not sample where the species in each pair meet, we cannot exclude the possibility of a narrow zone of hybridization. However, our data reveal little or no introgression away from the contact zone. These results, considered in light of previously published sequence evidence and divergence time estimates, allow an informed discussion of speciation in the region and taxonomy of the focal taxa.

### Speciation theory

Our results have implications for two aspects of speciation theory in Sundaland (Sheldon, Lim & Moyle, 2015) and in complex landscapes in general: timing and process. The apparent lack of gene flow between pairs of closely related taxa, combined with information on mtDNA divergences and/or molecular clock estimates in each group, provides insight into the potential timescale of speciation in certain Sundaic forest birds. All three of the target species pairs are separated by ca. 4.5% divergence in the ND2 gene, and the phylogenetic split between *Chloropsis kinabaluensis* and *Chloropsis cochinchinensis* has been estimated as early Pleistocene (Moltesen et al., 2012). Thus, a recent timeframe for diversification (e.g., during the last glacial maximum [LGM], 18,000–21,000 years ago) cannot be invoked in any of the taxa. Rather, the early Pleistocene (perhaps 2 Ma) is most reasonable for all three based on mtDNA divergences. The timing of secondary contact is also uncertain. Lowland samples from Western Borneo are only slightly differentiated genetically from samples collected in Western Sundaland (i.e., Sumatra and the Malay Peninsula; Moltesen et al., 2012; Moyle et al., 2005, 2011). This similarity implies recent range expansion, or possibly older range expansion with recurrent genetic homogenization of lowland taxa during recent glacial maxima. Habitat modeling and botanical studies indicate that Borneo, Sumatra, and the Malay Peninsula were united by suitable habitat for the study species during the LGM and perhaps the previous glacial event (Cannon, Morley & Bush, 2009; Raes et al., 2014), allowing movement of bird populations back and forth (Lim et al., 2011). A lack of genetic variation among isolated montane populations of *Enicurus borneensis* also argues for recent elevational displacement and allopatry.

The origin of montane species diversity in the Greater Sundas has been addressed explicitly by relatively few studies. Phylogenetic patterns and molecular dating in squirrels (*Sundasciurus*) indicated an old (Miocene or Pliocene) origin of montane clades and subsequent (Pliocene) diversification among montane regions (den Tex et al., 2010), and thus no influence from Pleistocene sea level and habitat changes. Barbets (*Megalaima*) display a different pattern, with montane endemics from each island related to widespread lowland species, rather than other montane taxa (den Tex & Leonard, 2013). However, divergence times for lowland–montane disjunctions span the Pliocene and Pleistocene, indicating that multiple historical factors might be involved in these speciation events. Our data support a role for Pleistocene (or possibly late Pliocene) isolation followed by more recent secondary contact resulting in elevational displacement in producing some of the diversity in Sundaland. The disparate conclusions of different studies are not surprising; the montane avifauna of the Sunda Region is complex, and a survey of its component species indicated that multiple processes at different time scales likely contributed to this diversity (Merckx et al., 2015; Sheldon, Lim & Moyle, 2015). As an additional caveat, it must be noted that any discussion of the timing of speciation events is based on highly uncertain molecular clock calibrations, because no fossils of rainforest birds exist from the region (Meijer, 2014), and must be considered with due caution.

Assuming a model of elevation parapatry similar to Diamond’s (1973), which is consistent with our data, *Enicurus leschenaulti* has progressed the furthest geographically, with complete elevational segregation across Borneo. Layered on top of—or more accurately, underneath—this elevational pattern, is another phylogeographic pattern that is seen in several other species (Lim et al., 2010, 2011; Sheldon et al., 2009). Lowland *Enicurus leschenaulti* from the western part of the island (Sarawak) are distinct from those in the Northeast (Sabah), and more similar to those in Sumatra (Moyle et al., 2005). This differentiation could be incipient stages of the same regional process (stage 2; Diamond, 1973, Fig. 8) that produced the focal species pairs.

### Alternative hypotheses

An origin of the Bornean montane species via long-distance dispersal from other montane areas in the region is a possible alternative to the elevational displacement hypothesis we propose here. Long-distance dispersal seems to have occurred in some other Bornean montane groups, such as the Island Thrush, *Turdus poliocephalus*, and some Himalayan taxa, including *Garrulax*, *Yuhina*, *Seicercus*, *Phylloscopus*, and *Pycnonotus flavescens* (Sheldon, Lim & Moyle, 2015). However, phylogenetic evidence renders this possibility unlikely for our study taxa for several reasons. First, these three species complexes contain no other montane endemic taxa, so dispersal among montane areas would also require subsequent extinction of the founder populations. Second, the montane species on Borneo are not sister to isolated lowland populations on any other island or the Asian mainland, so any long-distance dispersal mechanism is unlikely. In both *Arachnothera* and *Chloropsis*, the Bornean montane species is sister to a widespread clade of lowland subspecies, including those from lowland Western Borneo (Moltesen et al., 2012; Moyle et al., 2011). Relationships are less resolved in *Enicurus*, with the Bornean montane endemic, a clade of lowland Sundaic populations, and a Javan population in a polytomy (Moyle et al., 2005).

Distribution patterns produced by historical influences might be similar to those produced by recent ecological factors (Endler, 1982). In the current study system, ecological differences along elevational gradients (Slik et al., 2009) might be expected to provide strong selection that could induce a cessation of gene flow and parapatric speciation (Doebeli & Dieckmann, 2003), resulting in a similar pattern to that observed in the three pairs of birds. However, aspects of our study indicate that this is unlikely. Two of the species pairs—*Chloropsis* and *Arachnothera*—show elevational parapatry across only a portion of their distributions. In Northeast Borneo (i.e., Sabah), a single representative of each pair occurs (*Chloropsis kinabaluensis* and *Arachnothera everetti*). *Arachnothera everetti* spans from 200 m in the lowlands to 2,200 m in high mountains (Moyle et al., 2011). It is hard to imagine a selective agent that is strong enough to induce population subdivision in these species along an elevational gradient in Southwestern Borneo, but is so localized that it causes no discernable influence on the species’ distributions in Northeastern Borneo where the mountains are higher. Instead, it seems that the lowland taxa *Arachnothera modesta* and *Chloropsis cochinchinensis* simply have not yet reached Northeast Borneo, a scenario that again supports the recent invasion of *Arachnothera modesta* from Western Sundaland. The third species pair (*Enicurus leschenaulti*, *Enicurus borneensis*) has complete elevational parapatry across its range in Borneo, and so might be consistent with isolation along the elevational environmental gradient, but this distribution includes isolated montane areas (e.g., Gunung Kinabalu and Trus Madi), that contain genetically identical populations of the montane form (*Enicurus borneensis*). It is hard to imagine selection that would produce identical montane populations of *Enicurus borneensis*, unless admixture occurred during periods of cooler climate, which invokes historical influences. Despite much investigation, evidence of ecological parapatric divergence in birds is exceedingly rare (Fuchs, Fjeldså & Bowie, 2011; Smith et al., 2005, 2011), and our study offers no further evidence of it.

Extensive elevational replacement of congeners has been noted among plants on Borneo’s highest mountain, Mt. Kinabalu (Barkman & Simpson, 2001; Steenis, 1964). This “centric” portion of the montane endemic flora (vs. “eccentric” species whose closest relatives occur in other regions) is postulated to have evolved directly from lowland populations colonizing novel habitats as the mountains formed. Our data offer another source for putative centric montane endemics that is uncoupled from initial orogeny—allopatric speciation among islands in the lowlands of the region followed by elevation displacement upon secondary contact. This hypothesis is consistent with a broad synthesis of phylogenetic patterns that revealed many centric, montane endemics are younger than uplift of Mt. Kinabalu (Merckx et al., 2015).

### Taxonomy

Each of the three pairs of species has been considered to exhibit only subspecific variation by some taxonomists. Considered together with previously published data (Moltesen et al., 2012; Moyle et al., 2005, 2011), our results show unequivocally that each of the focal populations should be considered species. Each is morphologically diagnosable, has an independent evolutionary trajectory, is monophyletic, and shows little or no evidence of gene flow with its nearest relative despite ample opportunity for inter-breeding. However, it should be noted that morphological differences between species are small; indeed, previous reservation about the species status of these taxa relied largely on their lack of substantial morphological differentiation (Collar & Pilgrim, 2007). In the context of the proposed tempo and mode of diversification, the marginal morphological differentiation is not that unusual. Other studies have shown that elevational differences between recently diverged species generally evolve before differences in body size or feeding ecology (Kennedy et al., 2012; Price et al., 2014; Richman & Price, 1992). More broadly, these results indicate that any species concept that attempts to predict interbreeding potential simply on the basis of perceived morphological differences (Tobias et al., 2010) likely underestimates species diversity.

## Supplemental Information

10.7717/peerj.3335/supp-1 Supplemental Information 1 Supplementary figures and tables.

## Additional Information and Declarations

### Field Study Permissions

The following information was supplied relating to field study approvals (i.e., approving body and any reference numbers): Malaysia Prime Minister’s Department, UPE: 40/200/19 SJ.1039 and UPE: 0/200/19/2401.

### DNA Deposition

The following information was supplied regarding the deposition of DNA sequences: Hosner, Peter (2016): A genome wide assessment of stages of elevational parapatry in Bornean passerine birds reveals no introgression: implications for processes and patterns of speciation. Figshare. https://doi.org/10.6084/m9.figshare.3205750.v1

### Data Availability

For details on the individuals sampled, see Table 1.
