# Supplementary material for: A genome-wide assessment of stages of elevational parapatry in Bornean passerine birds reveals no introgression: implications for processes and patterns of speciation
Source: PeerJ. 2017 May 18;5:e3335. doi: 10.7717/peerj.3335 (PMC5438588; doi:10.7717/peerj.3335)
Supplement: Supplemental Information 1 [file peerj-05-3335-s001.pdf]

A genome-wide assessment of stages of elevational parapatry in Bornean passerine birds reveals no introgression: implications for processes and patterns of speciation

Supplementary Tables and Figures

Supplementary Figure 1. Number of SNPs per locus in the *Arachnothera* dataset.

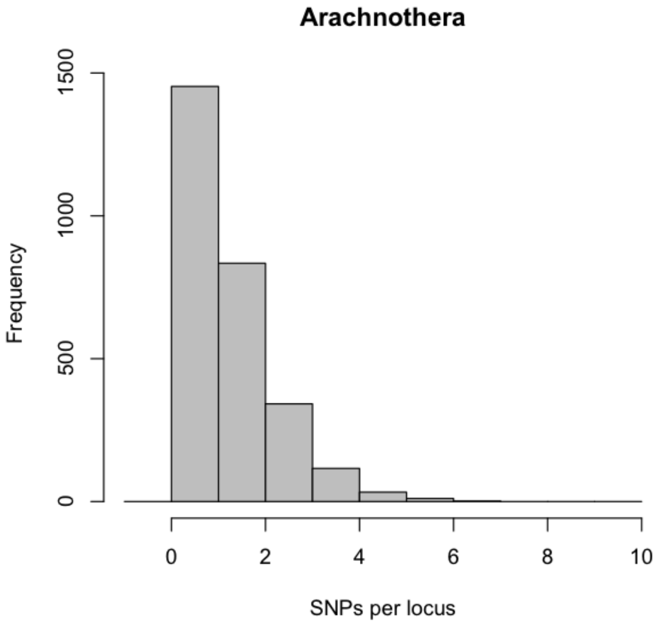

Supplementary Figure 2. Number of SNPs per locus in the *Chloropsis* dataset.

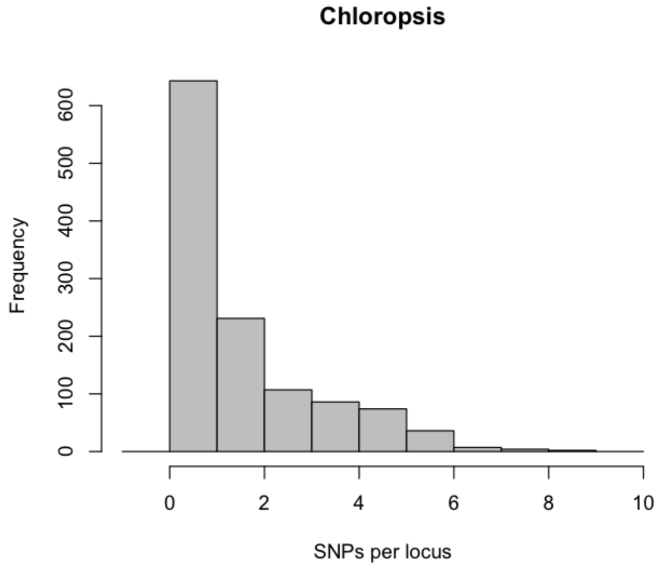

Supplementary Figure 3. Number of SNPs per locus in the Enicurus dataset.

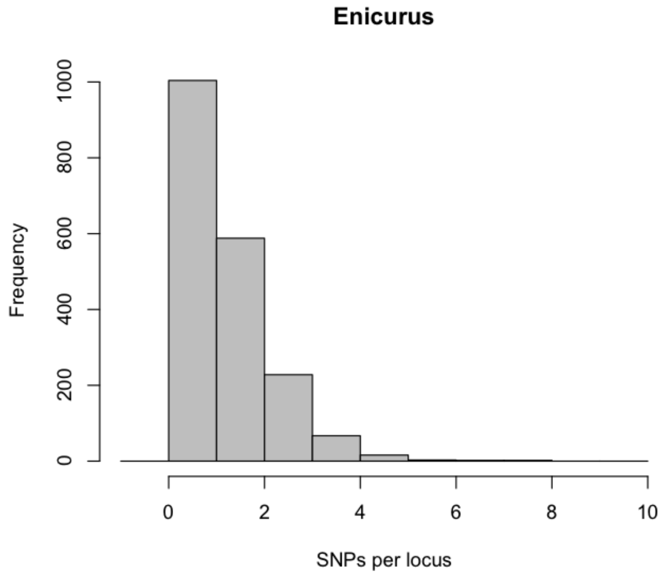

Supplementary Figure 4. Variation in population genetic differentiation (F_{ST}) between highland and lowland populations of each taxon using different SNP datasets. The parameters that varied in SNP calling were N (number of mismatches allowed between individuals when creating stacks) and m (minimum stack depth for a locus and associated SNPs to be included). The restrictive dataset used the settings [N5, m5, and tested for physical linkage and selection (see main manuscript)].

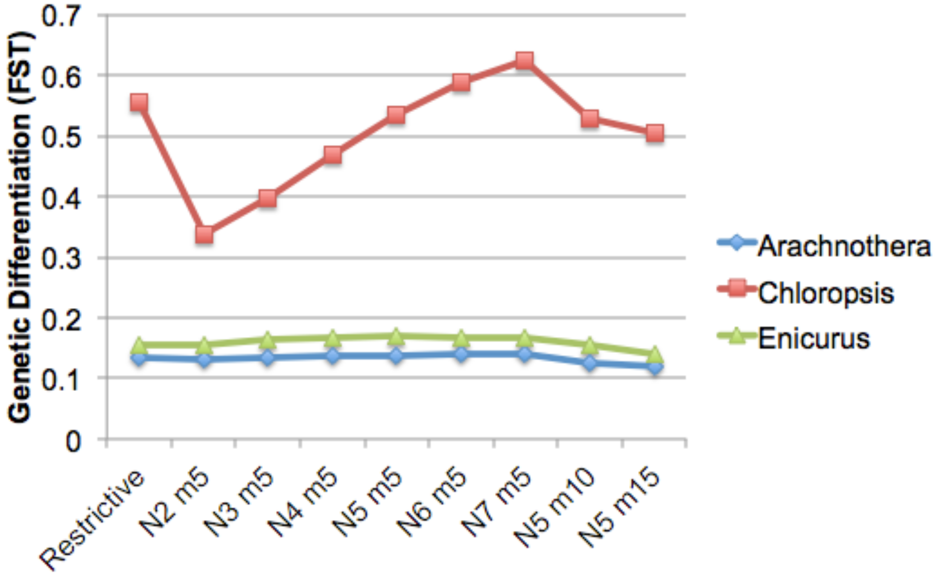

Supplementary Figure 5. Patterns of polymorphisms in *Arachnothera* with the different datasets as described in Supplementary Figure 4.

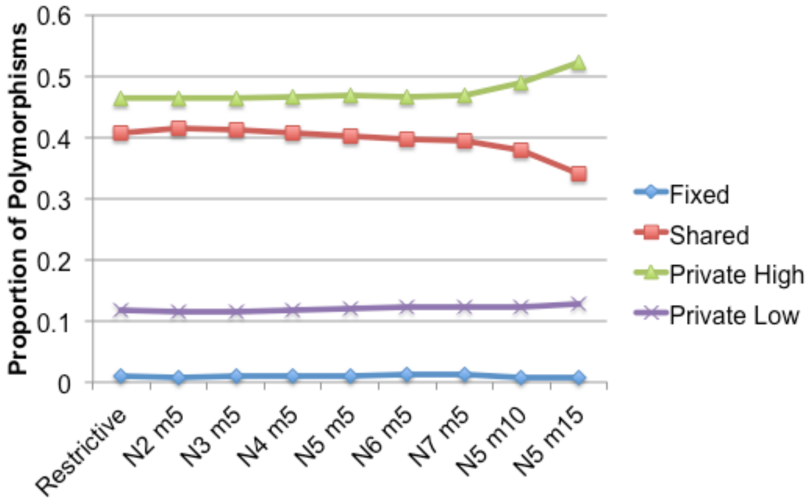

Supplementary Figure 6. STRUCTURE results for *Arachnothera* in the nine datasets described in Supplementary Figure 4. Shown are the assignment probabilities of individuals to the highland group. Open circles are the values presented in the main manuscript, with all other datasets represented by smaller closed circles.

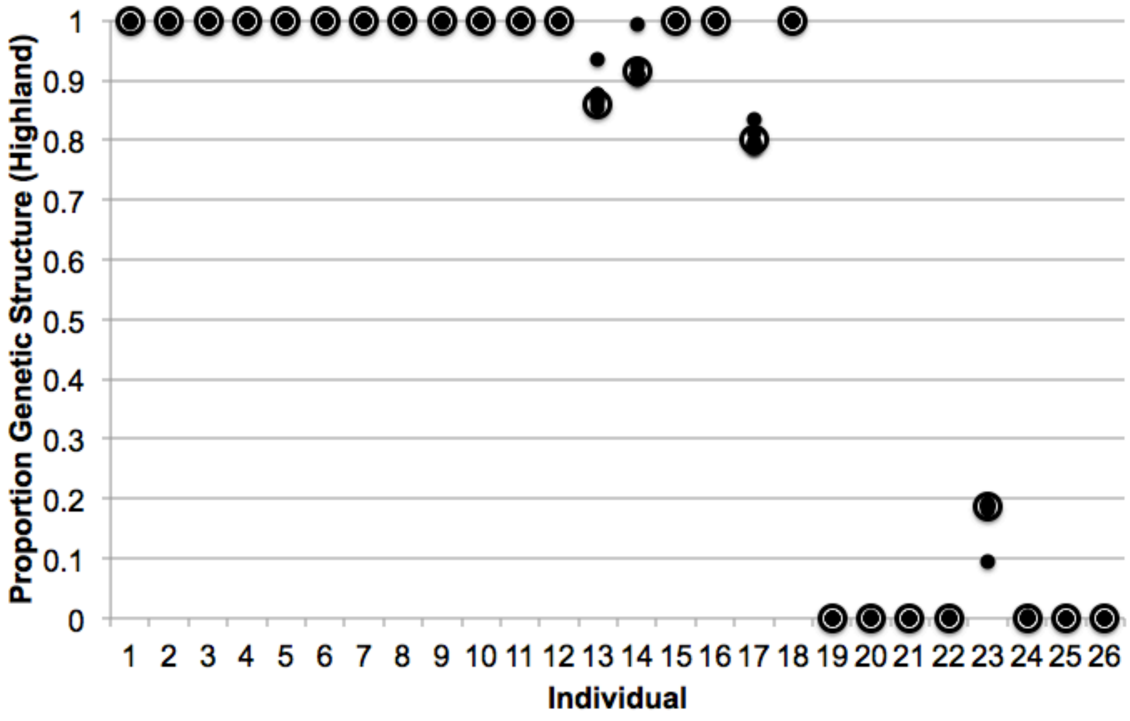

Supplementary Figure 7. Patterns of polymorphisms in *Enicurus* with the different datasets as described in Supplementary Figure 4.

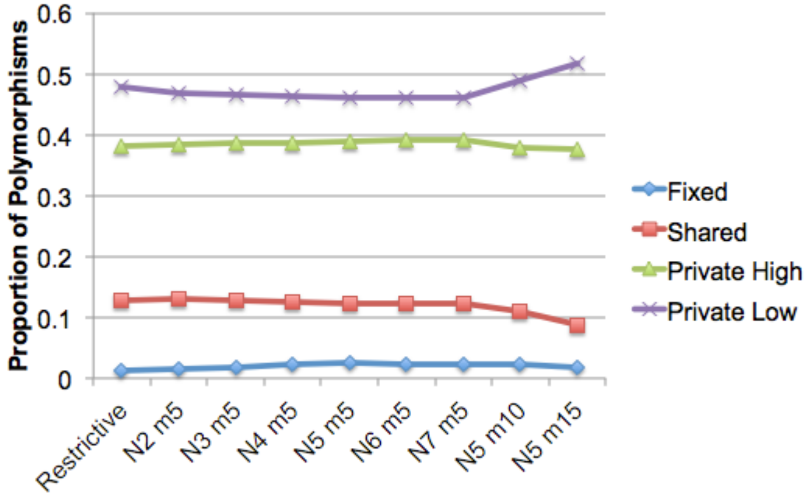

Supplementary Figure 8. STRUCTURE results for *Enicurus* in the nine datasets described in Supplementary Figure 4. Shown are the assignment probabilities of individuals to the highland group. Open circles are the values presented in the main manuscript, with all other datasets represented by smaller closed circles. The individuals with the largest variance in results (9, 12, and 14) were changes when the minimum stack depth (m) was changed to 15, resulting in far fewer SNPs (~150) and indicating less power to differentiate some of the individuals.

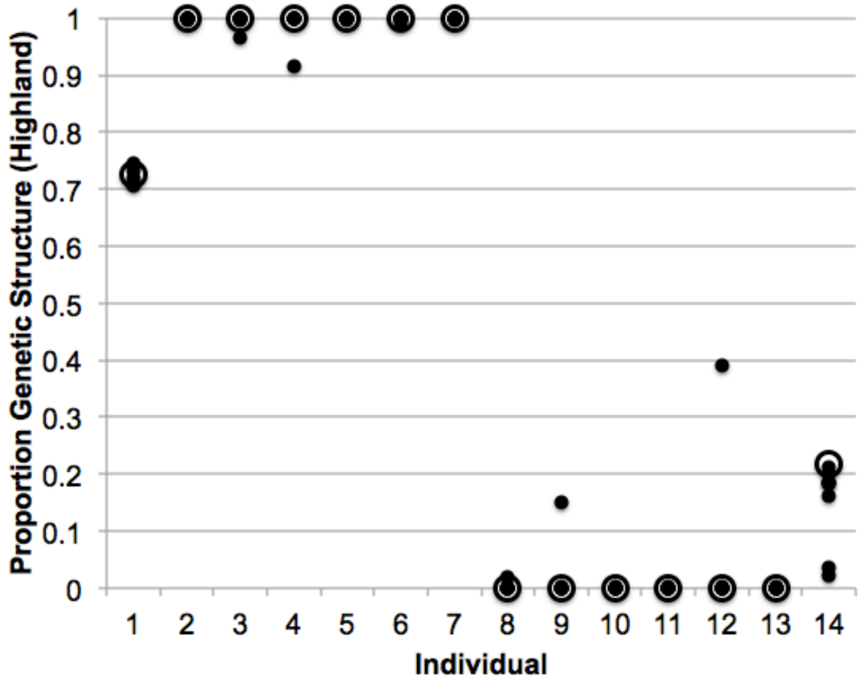

Supplementary Figure 9. Patterns of polymorphisms in *Chloropsis* with the different datasets as described in Supplementary Figure 4.

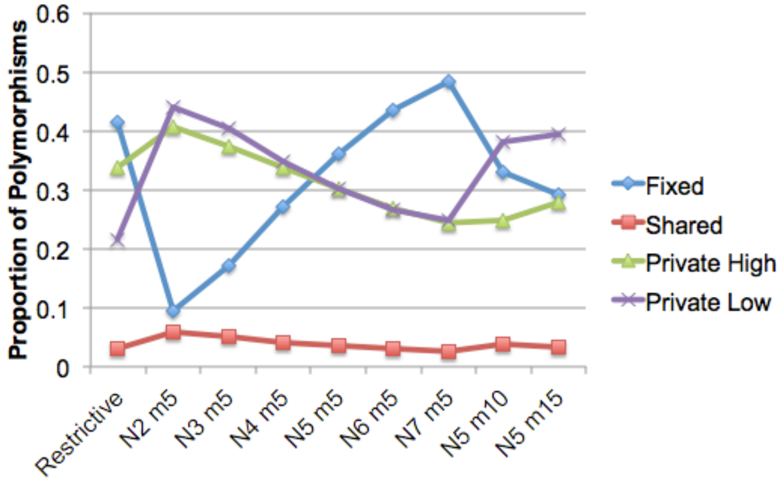

Supplementary Figure 10. STRUCTURE results for *Chloropsis* in the nine datasets described in Supplementary Figure 4. Shown are the assignment probabilities of individuals to the highland group. Open circles are the values presented in the main manuscript, with all other datasets represented by smaller closed circles. Two individuals (4 and 5) showed high variation in results across datasets.

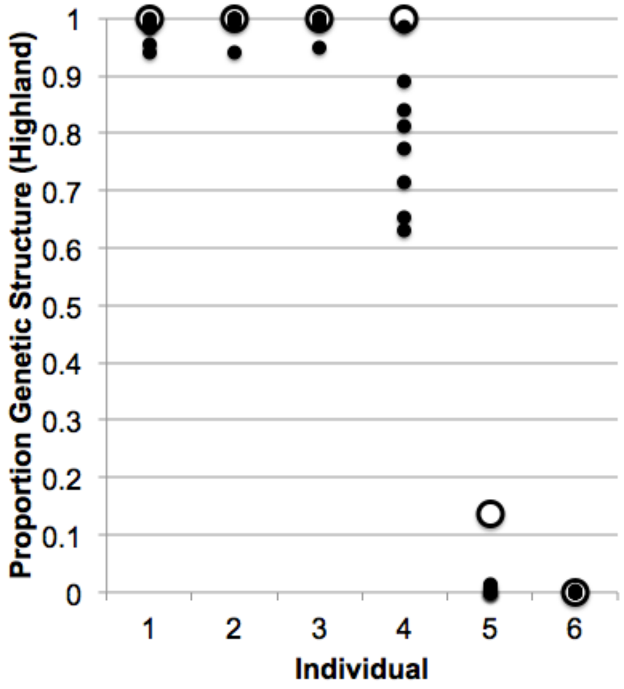

Supplementary Figure 11 – Log probability (blue) and delta K (red) for *Arachnothera*. Low probabilities at k = 1 exclude it as a possibility for this species. This identified k = 2.

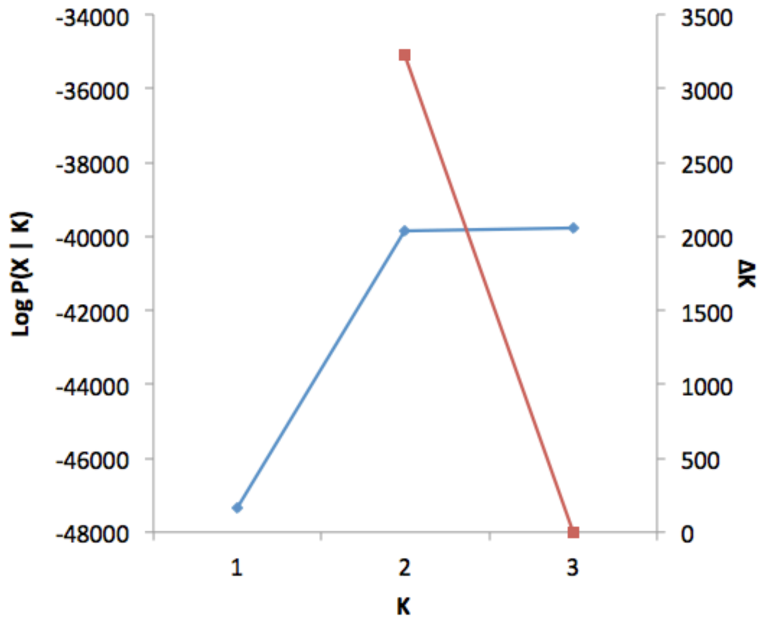

Supplementary Figure 12 – Log probability (blue) and delta K (red) for *Chloropsis*. Low probabilities at k = 1 exclude it as a possibility for this species. This identified k = 2.

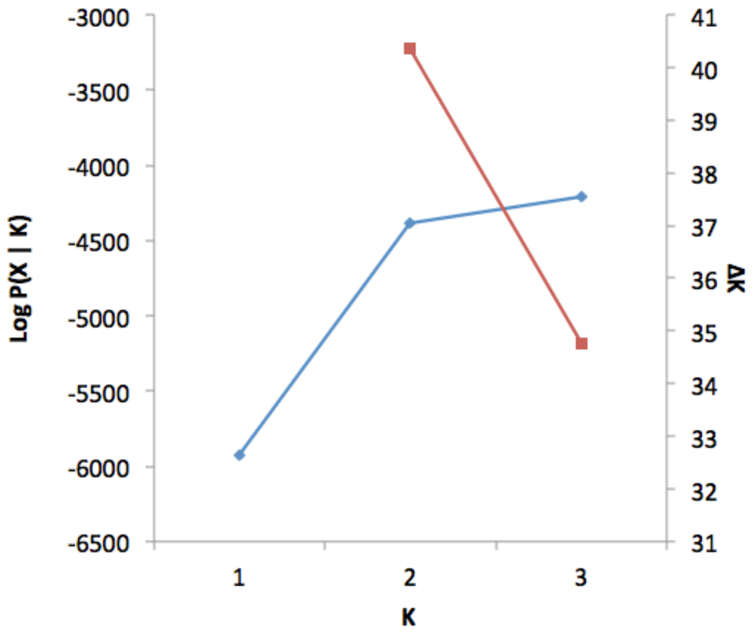

Supplementary Figure 13 – Log probability (blue) and delta K (red) for *Enicurus*. Low probabilities at k = 1 exclude it as a possibility for this species. This identified k = 2.

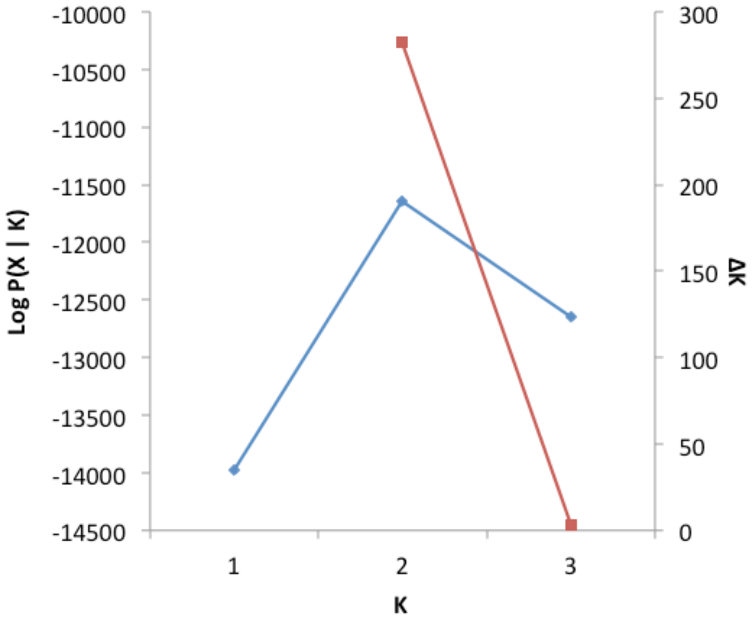

Supplementary Figure 14. Test to check if sample size was the driving force behind the high number of fixed differences in *Chloropsis*. Here, the *Arachnothera* and *Enicurus* datasets were subsampled (4 high elevation, 2 low) for 100 replicates, and their respective polymorphisms summarized. In no samples were the proportion of fixed differences similar to that seen in the empirical *Chloropsis* dataset.

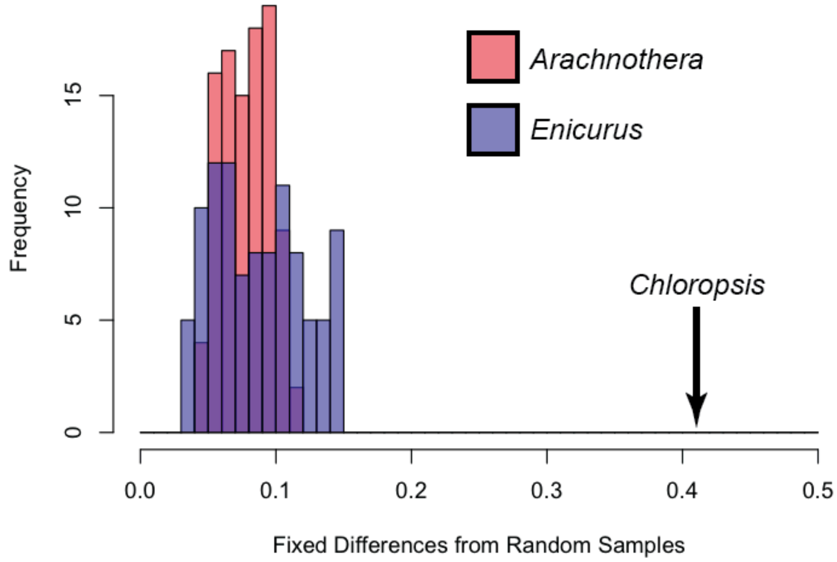

Supplementary Table 1. Results of three replicate runs of Migrate-N. All values are formatted as high elevation/low elevation values. Theta values are multiplied by 100.

	Median	Lower 95%	Upper 95%
Arachnothera			
Theta 1	0.353/0.287	0.013/0.000	0.667/0.560
Theta 2	0.367/0.300	0.013/0.000	0.667/0.560
Theta 3	0.393/0.447	0.013/0.000	0.667/0.560
2Nm 1	0.120/0.103	0.004/0.000	0.233/0.208
2Nm 2	0.131/0.101	0.005/0.000	0.243/0.196
2Nm 3	0.131/0.101	0.005/0.000	0.242/0.201
Chloropsis			
Theta 1	0.300/0.287	0.000/0.000	0.573/0.547
Theta 2	0.300/0.287	0.000/0.000	0.560/0.547
Theta 3	0.300/0.287	0.000/0.000	0.573/0.547
2Nm 1	0.098/0.096	0.000/0.000	0.193/0.187
2Nm 2	0.095/0.093	0.000/0.000	0.181/0.183
2Nm 3	0.102/0.098	0.000/0.000	0.201/0.191
Enicurus			
Theta 1	0.260/0.260	0.000/0.000	0.520/0.520
Theta 2	0.260/0.260	0.000/0.000	0.520/0.520
Theta 3	0.260/0.260	0.000/0.000	0.520/0.507
2Nm 1	0.091/0.090	0.000/0.000	0.185/0.184
2Nm 2	0.088/0.089	0.000/0.000	0.181/0.180
2Nm 3	0.090/0.092	0.000/0.000	0.186/0.183

Supplementary Table 2. Two-population dadi demographic modeling output. Abbreviations: arach = *Arachnothera*, enic = *Enicurus*, nu1 = population size of pop1 (relative to ancestral), nu2 = population size of pop2 (relative to ancestral), T = time since divergence, m12 = migration rate from pop2 into pop1, and m21 = migration rate from pop1 into pop2.

Pop1	Pop2	Model	Replicate	Composite Likelihood	AIC	Theta scaled (=4NeuL)	nu1	nu2	T	m12	m21
arach high	arach low	No divergence	1	-3574.78	7151.56	940					
arach high	arach low	No divergence	2	-3574.78	7151.56	940					
arach high	arach low	No divergence	3	-3574.78	7151.56	940					
arach high	arach low	Split, then isolation with migration	1	-336.27	684.54	324.6	4.96	1.1	1.69	0.15	0.33
arach high	arach low	Split, then isolation with migration	2	-332.73	677.46	336.6	4.97	1.27	1.45	0.15	0.29
arach high	arach low	Split, then isolation with migration	3	-340.86	693.72	344.1	4.98	0.91	1.55	0.17	0.48
arach high	arach low	Split, then no migration	1	-484.03	976.06	518.9	4.4	1.02	0.52		
arach high	arach low	Split, then no migration	2	-484.03	976.06	518.9	4.4	1.01	0.52		
arach high	arach low	Split, then no migration	3	-484.04	976.08	519.4	4.36	1.02	0.52		
enic high	enic low	No divergence	1	-2689.3	5380.6	928.4					
enic high	enic low	No divergence	2	-2689.3	5380.6	928.4					
enic high	enic low	No divergence	3	-2689.3	5380.6	928.4					
enic high	enic low	Split, then isolation with migration	1	-272.13	556.26	275.3	2.17	4.89	2.16	0.15	0.04
enic high	enic low	Split, then isolation with migration	2	-280.49	572.98	291.3	1.75	4.99	2.04	0.22	0.07
enic high	enic low	Split, then isolation with migration	3	-278.51	569.02	270.4	2.16	4.93	2.23	0.16	0.02
enic high	enic low	Split, then no migration	1	-324.9	657.8	342.7	2.28	4.96	1.3		
enic high	enic low	Split, then no migration	2	-323.61	655.22	349.9	2.16	4.92	1.26		
enic high	enic low	Split, then no migration	3	-335.99	679.98	374.9	1.62	4.99	1.18		
